# Efficacy of Transcutaneous Vagus Nerve Stimulation to Suppress Paroxysmal Atrial Fibrillation: A Systematic Review

**DOI:** 10.7759/cureus.86435

**Published:** 2025-06-20

**Authors:** Carlos Alberto Umaña Mejia, Carolina S Ascencio Flores, Selene G Gudiño Radilla, Myles A Laurence Salinas, Diego A Moreno Alvarez, Eduardo Corona Vargas, Mara Durán Borbolla, José M Morales Barajas, David Jiménez Frutos, Gerardo-Anuar Molinar Perez, Ricardo Gonzalez, John Manuel Dorado Ramírez, Martin Gomez-Lujan, Jaqueline L Castillo, Mauricio Montelongo Quevedo, Jose R Flores Valdés

**Affiliations:** 1 General Medicine, Universidad Autonoma De Guadalajara, Guadalajara, MEX; 2 Centro Universitario de Ciencias de la Salud, Universidad de Guadalajara, Guadalajara, MEX; 3 General Medicine, Universidad Autonoma de Guadalajara, Guadalajara, MEX; 4 Cardiology, Instituto Nacional de Cardiologia "Ignacio Chavez", Mexico City, MEX; 5 Faculty of Health Sciences, Universidad Anahuac Mexico, Mexico City, MEX; 6 Faculty of Medicine and Biomedical Sciences, Universidad Autonoma de Ciudad Juarez, Juárez City, MEX; 7 Faculty of Medicine, Universidad del Desarrollo, Concepcion, CHL; 8 Internal Medicine, Instituto Mexicano del Seguro Social, Mexico City, MEX; 9 Faculty of Medicine, Universidad Nacional Federico Villareal, Lima, PER; 10 General Medicine, Universidad Autónoma de Guadalajara, Guadalajara, MEX; 11 Research, Oncology Consultants, PA, Houston, USA

**Keywords:** arrhythmia suppression, atrial fibrillation burden, inflammatory markers, low-level transcutaneous vagus nerve stimulation, paroxysmal atrial fibrillation

## Abstract

Paroxysmal atrial fibrillation (PAF) is a common cardiac arrhythmia marked by episodic, irregular heart rhythms, contributing significantly to both morbidity and mortality. Traditional management typically involves antiarrhythmic medications and catheter ablation; however, novel, non-invasive approaches, such as low-level transcutaneous vagus nerve stimulation (LLTS), are emerging as promising alternatives. LLTS aims to modulate autonomic nervous system activity through vagus nerve activation, with potential benefits, including the suppression of arrhythmias and a reduction in systemic inflammation.

To evaluate the effectiveness of LLTS in patients with PAF, a systematic review was conducted according to the Preferred Reporting Items for Systematic Reviews and Meta-Analyses (PRISMA) guidelines. Searches were performed in the PubMed and ScienceDirect databases covering literature from 2015 to 2024. The review included randomized controlled trials (RCTs), cohorts, and case-control studies that evaluated adult patients with PAF undergoing LLTS interventions. Three RCTs met the inclusion criteria, involving a total of 121 participants who received either LLTS or sham stimulation. LLTS and sham stimulation were standardized among studies and adequately blinded. Outcomes assessed across studies included suppression of arrhythmia episodes, changes in heart rate variability, levels of inflammatory markers, and overall reduction in atrial fibrillation (AF) burden during a six-month follow-up period.

Results showed that LLTS significantly reduced AF burden, the time a patient spends in AF, compared to sham stimulation, with reductions of up to 85% observed at six months. Patients receiving LLTS also exhibited lower levels of inflammatory markers, such as tumor necrosis factor-alpha, indicating an anti-inflammatory effect. Importantly, no major adverse events were reported in any of the included trials. However, limitations of the current evidence include small sample sizes, homogeneity in the dose and timing of stimulation (20 Hz for one hour), follow-up time of six months, lack of continuous ECG monitoring, and variability in individual patient responses to LLTS.

In conclusion, LLTS appears to be a safe and effective adjunctive treatment option for PAF, with significant potential for reducing AF burden and inflammation. Although early results are promising, further research involving larger cohorts and longer follow-up periods is necessary to confirm its long-term benefits and identify patient populations that may derive the greatest benefit from this therapy.

## Introduction and background

Atrial fibrillation (AF) is a supraventricular arrhythmia characterized by uncoordinated electrical activity and deterioration of proper atrial mechanical function, with an irregular ventricular response. There are various types of AF, among which paroxysmal atrial fibrillation (PAF) is defined as several episodes, lasting ≤7 days, often self-terminating within 24 hours [1]. Focal activity and reentry circuits are two key electrophysiological mechanisms implicated in the onset and maintenance of AF. Among the most recognized sources of focal activity are the pulmonary veins, making pulmonary vein isolation the cornerstone intervention for managing PAF. Emerging evidence highlights the role of reactive oxygen species in AF, with NADPH oxidases (NOX) and mitochondrial pathways identified as primary sources of oxidative stress in cardiac tissue. Abnormal activation of NOX enzymes and mitochondrial impairment have both been linked to AF development and progression [2].

PAF can be asymptomatic. The symptoms vary depending on heart rate, duration of AF, underlying cardiac function, and the patient’s perceptions. The most common symptoms include palpitations, chest pain or discomfort, shortness of breath, fatigue, or lightheadedness. Occasionally, the patient may initially present with heart failure symptoms or an embolic event. Syncope is a rare presentation that indicates a form complicated by the presence of sinus node dysfunction, aortic stenosis, cerebrovascular disease, or an accessory pathway that, in some cases, allows for extremely rapid atrioventricular conduction. The basic paraclinical examinations include an electrocardiogram (ECG), chest X-ray, transthoracic echocardiography, and thyroid function tests, particularly in cases of newly diagnosed or difficult-to-control AF. Additional investigations may be indicated for certain patients, including stress testing (AF during exertion, detection of myocardial ischemia), rhythm Holter monitoring (to diagnose suspected AF or evaluate treatment effectiveness), transesophageal echocardiography (for stroke evaluation or before cardioversion), and electrophysiological studies [3].

In the United States alone, at least 3 to 6 million people have AF, and the numbers are projected to reach ≈6 to 16 million by 2050 [4]. It is an important risk factor for ischemic stroke, resulting in a fivefold increased stroke risk and a twofold increased mortality [5]. The high prevalence observed and its increased mortality clearly show that there is a need for alternative treatments to be implemented to reduce AF. Currently, the management approach for patients with PAF focuses on controlling the arrhythmia during episodes and implementing maintenance treatment to prevent their recurrence, as well as to reduce the risk of stroke or systemic thromboembolism.

The present research aims to explore new therapeutic options, particularly low-level transcutaneous vagus nerve stimulation (LLTS), which could offer a promising alternative for treating paroxysmal AF. This method leverages the vagus nerve’s effect on the heart’s electrical activity and its influence on the balance of the autonomic nervous system. The research emphasizes several methodologically comparative questions regarding the treatment of this pathology: Does the stimulation modality offer reductions in AF burden, does it affect inflammatory pathways such as NOX or biomarkers such as TNF-α, and are there specific patient groups who would benefit more? Additionally, what are the potential complications associated with this treatment? Addressing these questions is crucial to evaluating the effectiveness and safety of this innovative approach.

## Review

Methodology

This study followed the Preferred Reporting Items for Systematic Reviews and Meta-Analyses (PRISMA) 2020 guidelines and previous literature to ensure a structured, transparent, and comprehensive review process [6,7]. Following the registration, secured and backed up by the PROSPERO database with the registration number CRD42024621582.

Search Methods

To identify relevant literature, a thorough search was performed using the PubMed and ScienceDirect databases. Both Medical Subject Headings (MeSH) terms and carefully selected keywords were utilized to capture studies pertinent to the research focus. A strict set of inclusion and exclusion criteria was applied to ensure only high-quality and directly relevant studies were considered. Articles that lacked full text access or could not be retrieved through interlibrary loan services were omitted. The study selection process followed the structure outlined in the PRISMA flow diagram [6], providing transparency and reproducibility. This methodical search and screening process resulted in a consistent and high-quality dataset, enabling a more robust and dependable analysis. Keywords used include “paroxysmal atrial fibrillation”, “adult”, “transcutaneous nerve stimulation”,” vagus nerve”

Types of Participants

A specific set of inclusion criteria was created that included individuals of both sexes, patients 18 years of age or older with PAF, documented by ECG, implantable device, or Holter monitor. We excluded studies involving patients with left ventricular dysfunction, significant valvular disorder (i.e., prosthetic valve or hemodynamically relevant valvular diseases), stroke or myocardial infarction, in addition, patients with sick sinus syndrome, second or third degree atrioventricular (AV) block, bifascicular block and prolonged first degree AV block (PR > 300 ms), in the absence of a pacemaker, recurrent vasovagal syncopal episodes, unilateral or bilateral vagotomy, pediatric populations, pregnancy or nursing.

Types of intervention

This systematic review focuses on therapy with a transcutaneous electrical nerve stimulation device, which delivers low-level transcutaneous electrical stimulation to the auricular branch of the vagus nerve at the tragus, while the control group had a matching placebo.

Types of Studies

In our investigation of transcutaneous vagus nerve stimulation for suppression of PAF, a rigorous search of PubMed and ScienceDirect was performed. References were collected from 2015 to September 2024. This systematic review included randomized controlled trials (RCTs), cohort, and case-control studies reporting on the efficacy of transcutaneous vagus nerve stimulation to suppress PAF. We excluded case reports, case series, cross-sectional studies, dissertations, book chapters, protocol articles, reviews, news articles, conference abstracts, letters to the editor, editorials, and comment publications. Literature outside the English or Spanish language is understood as a potential source of bias.

Type of Outcomes

The primary outcome of this systematic review is to evaluate the efficacy of arrhythmia suppression, including parameters such as the frequency of arrhythmia recurrence and duration of suppression. Secondary outcomes include variability in heart rate, inflammatory markers, and progression to permanent AF.

Selection of Studies, Data Extraction, and Screening

Two reviewers (EC, MD) independently screened titles and abstracts using Rayyan software [8]. A third reviewer (DM) subsequently evaluated the relevance of the identified studies based on pre-established inclusion and exclusion criteria. This was followed by a full-text screening, during which two reviewers (DM, JM) independently assessed the studies for eligibility using the same selection criteria. Any discrepancies that arose were addressed through discussion, with input from a third reviewer (SG). All records retrieved from the database searches were initially assessed for relevance, and those appearing to meet the eligibility criteria underwent a comprehensive review before being either included or excluded.

Data Evaluation: Assessment of Risk of Bias

The evaluation process was conducted in alignment with the *Cochrane Handbook for Systematic Reviews of Interventions* [9]. To assess methodological quality, the Cochrane Risk of Bias tool was applied to the included RCTs [10]. Two reviewers (MD, JD) independently evaluated the risk of bias for each study, adhering to the criteria set out in the respective tools. Any disagreements in assessments were addressed through discussion and resolved with the input of a third blinded reviewer (RG).

Results

A systematic examination of the literature was completed to assess the efficacy of LLTS vs. sham stimulation. Specifically, we focused on the efficacy of arrhythmia suppression, including parameters such as frequency of arrhythmia recurrence and duration of suppression. Secondary outcomes include variability in heart rate, inflammatory markers, and progression to permanent AF. Our search covered studies from 2015 to the present, with the use of two databases: PubMed and ScienceDirect.

 A systematic review's cornerstone is methodically identifying and selecting pertinent studies from an extensive pool of literature. Our search strategy began with an expansive database query yielding 311 articles. No duplicate articles were found. After a structured screening of titles and abstracts, 302 papers were excluded, and 9 publications were chosen for full-text evaluation. This multi-stage screening process resulted in the inclusion of three high-quality studies, each meeting the strict eligibility criteria established for this review.

**Figure 1:**
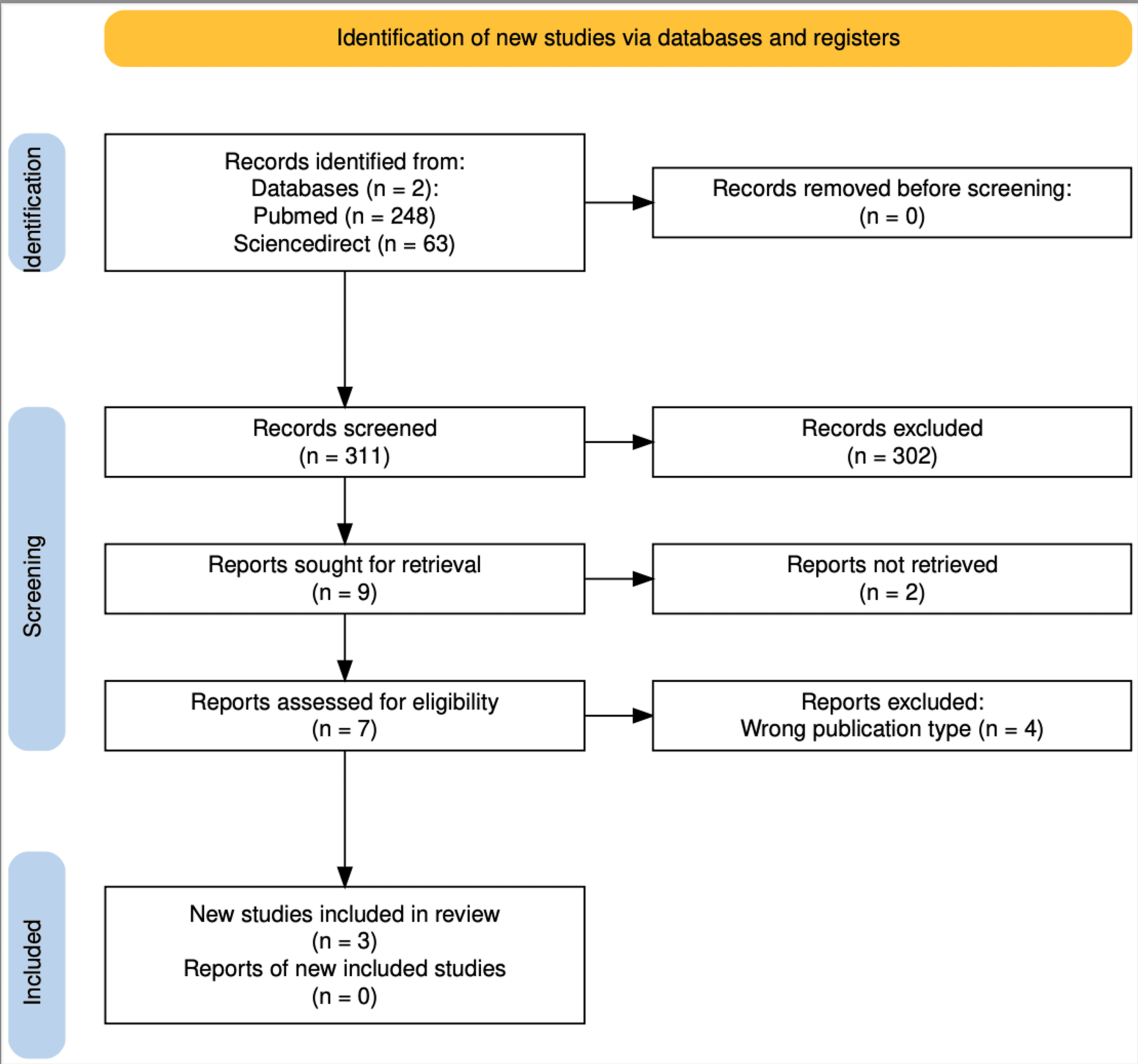
Flow diagram of bibliographic search (following PRISMA guidelines). Source: [6]. PRISMA, Preferred Reporting Items for Systematic Reviews and Meta-Analyses

Figure 1 presents a visual overview of the study selection pathway, structured according to the PRISMA flow diagram [6]. This sequential representation enhances the transparency of the selection process, clearly outlining how studies were filtered and finalized for inclusion.

The assessment of the risk of bias in the selected studies showed certain areas of methodological concern. First, the bias of the random allocation was rated as "some concerns" in all the studies that were chosen, which is interpreted as possible limitations in the presentation of the information or execution of the random allocation procedures, which would translate into selection bias.

In the bias due to the lack of outcome data, "some concerns" were also indicated, since the lack of data could affect the reliability of the findings, specifically if the loss of data was not random. The bias in the measurement of the results showed "some concerns," possibly reflecting problems or conflicts with the blinding of the results or other factors that influence the objectivity of the results.

The bias item in the reported outcome section was assessed as “low risk” for all studies, showing that the reported results were well aligned with the predefined analyses and were not selectively reported.

**Figure 2:**
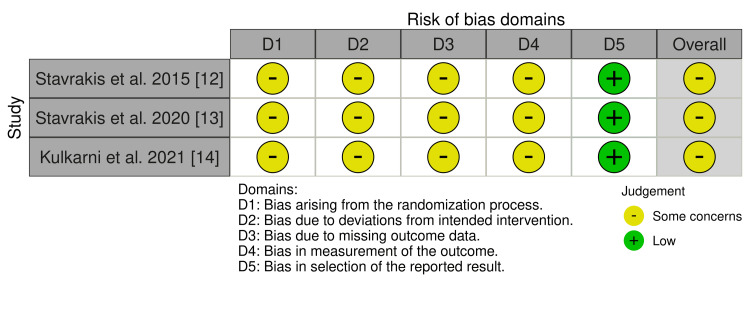
Risk of bias. Each article was assessed for risk of bias [10,11]. Of the three articles evaluated, all exhibited some concerns regarding overall bias [12-14].

**Figure 3:**
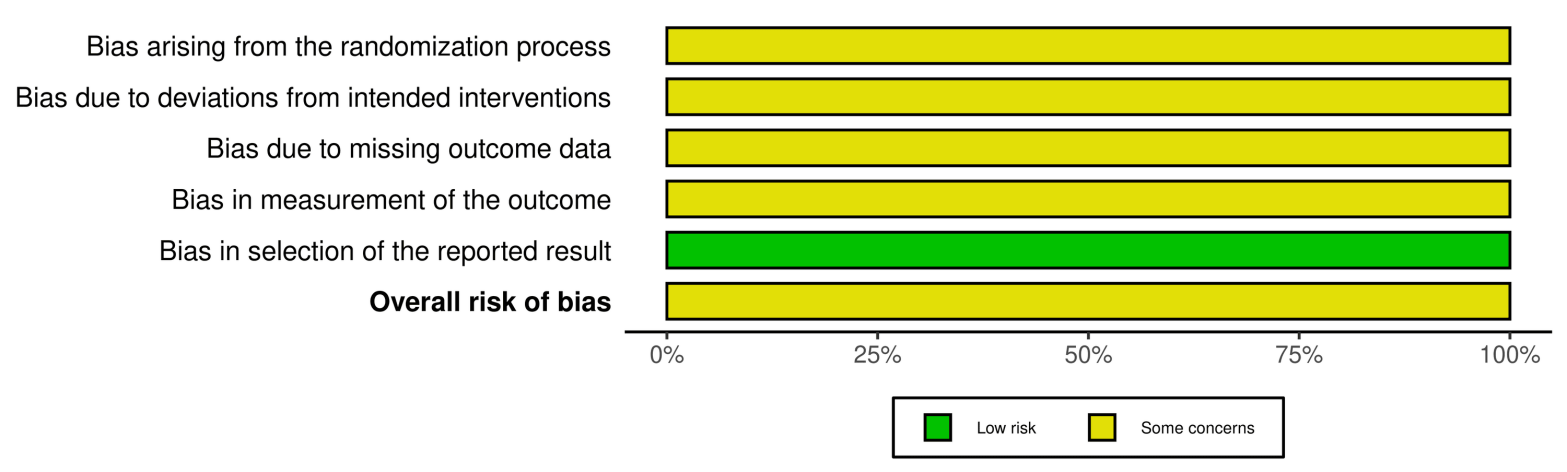
Summary plot for randomized controlled trials. This diagram [10,11] shows part of the risk of bias assessment [12,13,14].

Overall, the studies were rated as *some concerns* for risk of bias, indicating that while the main findings remain interpretable, caution should be exercised when concluding. Figures 2-3 visually demonstrate the assessment of risk of bias.

Patient Population

The characteristics of the patients enrolled in the three studies are summarized in Table 1. The three studies included a total of 121 patients; 59 were randomized to the sham group, and 62 to the active group. There were no significant differences in clinical characteristics between the sham and active groups.

**Table 1:** General outcomes. AF, atrial fibrillation; PWA, pulse wave alternans; Hz, hertz; LF/HF, low frequency/high frequency; F/M, female/male; IL, interleukin; TNF, tumor necrosis factor; CRP, c-reactive protein; LLTS, low-level transcutaneous vagus nerve stimulation; NA, not available; mo, months

Author (Year)	Stavrakis et al. (2015) [12]	Stavrakis et al. (2020) [13]	Kulkarni et al. (2021) [14]
Study type	Randomized controlled trial (RCT)	Randomized controlled trial (RCT)	Randomized controlled trial (RCT)
# of total patients in study	40 patients	53 patients	28 patients
# intervention group	20 patients	26 patients	16 patients
# sham group	20 patients	27 patients	12 patients
Hz level of intervention	20 Hz	20 Hz	20 Hz
Duration of stimulation or sham	1 hour	1 hour	1 hour
Mean age of the intervention group (years)	60.9 ± 7.8	65.2 ± 14.5	66.3 ± 7.6
Mean age of the sham group (years)	62.9 ± 9.8	68.0 ± 10.6	60.3 ± 12.5
# of F/M in the intervention group, *n* (%)	Male: 15 (75%); female: 5 (15%)	Male: 12 (46%); female: 14 (54%)	Male: 8 (50%); female: 8 (50%)
# of F/M in the sham group	Male: 11 (55%); female: 9 (45%)	Male: 12 (44%); female: 15 (56%)	Male: 6 (50%); female: 6 (50%)
F/M race or ethnic group, *n* (%) intervention group	NA	Non-White 1 (4%); White 25 (96%)	NA
F/M race or ethnic group, *n* (%) sham group	NA	Non-White 3 (11%); White 24 (89%)	NA
Cardiovascular disease history and risk factors (#) intervention group, *n* (%)	Hypertension: 15 (75%); diabetes: 1 (5%); coronary artery disease: 3 (15%); obstructive sleep apnea: 8 (40%)	Diabetes mellitus: 4 (15%); hypertension: 17 (65%); coronary artery disease: 5 (19%); heart failure: 6 (23%); obstructive sleep apnea: 5 (19%)	Diabetes mellitus: 3 (19%); hypertension: 12 (75%); coronary artery disease: 5 (31%); heart failure: 3 (19%); obstructive sleep apnea: 5 (31%)
Cardiovascular disease history and risk factors (#) sham group, *n* (%)	Hypertension: 15 (75%); diabetes: 5 (25%); coronary artery disease: 4 (20%); obstructive sleep apnea: 7 (35%)	Diabetes mellitus: 7 (26%); hypertension: 23 (85%); coronary artery disease: 7 (26%); heart failure: 5 (19%); obstructive sleep apnea: 9 (33%)	Diabetes mellitus: 3 (25%); hypertension: 9 (75%); coronary artery disease: 4 (33%); heart failure: 3 (25%); obstructive sleep apnea: 5 (42%)
Biomarkers	TNF-α; CRP; IL-6; IL-10	TNF-α; IL-6; IL-1b; IL-10; IL-17	NA
Follow-up time	6 months	6 months	6 months
# deaths in the intervention group	NA	1	NA
# deaths in the sham group	NA	2	1
Adverse events in the intervention group	No major adverse events were observed in the study.	No major adverse events were observed in the study.	No major adverse events were observed in the study.
Adverse events in the sham group	No major adverse events were observed in the study.	No major adverse events were observed in the study.	No major adverse events were observed in the study.
AF burden in the intervention group (%)	NA	Baseline: 4.3 (0.2-31.0); 3 mo: 2.0 (0.0-21.0); 6 mo: 2.0 (0.0-11.0)	Baseline: 14.3 ± 12.2; 3 mo: 12.7 ± 9.2; 6 mo: 10.3 ± 8.7
AF burden in the sham group (%)	NA	Baseline: 1.0 (0.0-15.0); 3 mo: 3.0 (0.1-11.0); 6 mo: 8.5 (0.0-42.0).	Baseline: 13.3 ± 13.1; 3 mo: 15.2 ± 13.9; 6 mo: 25.7 ± 16.6
PWA burden in the intervention group (%)	NA	NA	Baseline: 8.2 ± 7.7; 3 mo: 8.1 ± 6.0; 6 mo: 7.7 ± 3.5
PWA burden in the sham group (%)	NA	NA	Baseline: 9.6 ± 8.7; 3 mo: 11.2 ± 8.9; 6 mo: 13.9 ± 9.7
Change in AF duration in the intervention group	AF duration decreased by 6.3 ± 1.9 minutes	NA	NA
Change in AF duration in the sham group	AF duration increased by 1.4 ± 1.8 minutes	NA	NA
LF/HF ratio	NA	2.16	NA

In all intervention groups, the main risk factors identified were hypertension, diabetes mellitus, and coronary artery disease.

Effect of LLTS on Biomarkers

Stavrakis et al. [12] and Stavrakis et al. [13] used inflammation cytokines as biomarkers to evaluate the effects of the intervention. In the study by Stavrakis et al. [12], tumor necrosis factor-alpha (TNF-α), C-reactive protein (CRP), interleukin-6 (IL-6), and IL-10 were measured at baseline and one hour post-intervention. In contrast, the study by Stavrakis et al. [13] assessed TNF-α, IL-6, IL-1β, IL-10, and IL-17, with serum cytokine levels measured at baseline, three months, and six months.

In the study by Stavrakis et al. [12], no significant differences were observed in coronary sinus measurements. However, systemic TNF-α levels were significantly reduced in the active group (2.3 ± 0.3 pg/mL, *P* = 0.006) compared to baseline. Similarly, systemic CRP levels decreased significantly in the active group (1.9 ± 1.4 ng/L, *P* = 0.001). These changes were not observed in the control group.

Consistently, in the study by Stavrakis et al. [13], serum TNF-α levels were 23% lower in the active group compared to the control group (ratio of medians: 0.77; 95% confidence interval [CI]: 0.63-0.94; *P* = 0.0093). No significant differences were observed in the levels of the other cytokines.

In the study by Kulkarni et al. [14], pulse wave alternans (PWA) was described as a biomarker for evaluating the overall response to treatment; however, the results are discussed separately next.

Effect of LLTS on AF Burden

Two studies [13,14] reported changes in AF burden. In the study by Kulkarni et al. [14], results were compared at three and six months. The baseline AF burden was 13.0 ± 13.1 in the sham group (*P* < 0.05) and 14.3 ± 12.2 in the active group (*P* < 0.05). At the three-month follow-up, the burden increased to 15.2 ± 13.9 (*P *< 0.05) on the sham group and decreased to 12.7 ± 9.2 (*P *< 0.05) on the active group. Moreover, at the six-month follow-up, the burden increased to 25.7% ± 16.6% (*P *< 0.05) on the sham group and decreased to 10.3% ± 8.7% (*P* < 0.05) on the active group. Showing a significantly lower AF burden after six months of active chronic LLTS (*P* < 0.05) [14].

In the study by Stavrakis et al. [13], follow-up assessments were conducted at three and six months. In the sham group, the AF burden at baseline was 1.0% (0.0-15.0, *P* = 0.016), which increased to 3.0% (0.1-11.0, *P* = 0.016) at three months and further to 8.5% (0.0-42.0, *P* = 0.016) at six months. In the active group, the AF burden at baseline was 4.5% (0.2-31.0, *P* = 0.016), which decreased to 2.0% (0.0-21.0, *P* = 0.016) at three months and remained at 2.0% (0.0-11.0, *P* = 0.016) at six months. Similar results were found in both studies, considering that the median AF burden combining across the three- and six-month time points was reduced by 85% in the active group compared with the sham group (ratio of medians: 0.25; 95% CI: 0.08-0.77; *P* = 0.016) [13].

The included studies had small sample sizes, and their results should be interpreted with caution.

Effect of Chronic LLTS and Acute LLTS on PWA Burden

One study by Kulkarni et al. [14] reported changes in PWA burden, evaluating the effects of both acute and chronic active LLTS. Follow-up was conducted at three months to assess the acute intervention and at six months to assess the chronic intervention.

The baseline PWA burden was 9.6% ± 8.1% (*P* < 0.05) in the sham group and 8.2% ± 7.7% (*P* < 0.05) in the active group. At the three-month follow-up, the PWA burden increased to 11.2% ± 8.9% (*P *< 0.05) in the sham group, compared to 8.1% ± 6.0% in the active group. At the six-month follow-up, the PWA burden increased to 13.9 ± 9.7% (*P* < 0.05) in the sham group and decreased to 7.7% ± 3.5% (*P* < 0.05) in the active group. These findings demonstrate a significant reduction in PWA burden in the active group compared to the sham group after six months of chronic LLTS (*P* < 0.05) [14]. However, it is important to note that this study had a small sample size of 28 participants, and the results should be interpreted with caution.

Change in AF Duration

In the study by Stavrakis et al. [12], changes in pacing-induced AF duration were reported. In the intervention group, AF duration decreased by 6.3 ± 1.9 minutes, whereas in the control group, it increased by 1.4 ± 1.8 minutes, with a significant between-group difference (*P* = 0.002).

No major adverse events, such as myocardial infarction or stroke, were reported in any of the studies. Reported deaths were unrelated to the treatment and were attributed to unspecified causes.

Discussion

AF remains one of the most prevalent and challenging cardiac arrhythmias, significantly contributing to morbidity and mortality. As life expectancy increases globally, the incidence of AF is expected to rise, further highlighting the need for effective treatment strategies. Although current treatments, including antiarrhythmic drugs and catheter ablation, are widely used, AF continues to present a major burden on healthcare systems [4]. In this context, the role of LLTS in managing PAF has emerged as an area of considerable interest. The studies analyzed in this systematic review provide important insights into the efficacy of LLTS in patients with PAF compared to sham stimulation. The main objective of our review was to evaluate the efficacy of arrhythmia suppression utilizing parameters such as frequency of arrhythmia recurrence and duration of suppression. Three studies were included, with a combined population of 121 patients. All studies utilized 20 Hz stimulation for a duration of one hour. The mean age of the study population ranged from 60.9 to 68 years, limiting the evaluation of treatment effects in patients outside this age range.

Across two studies [13,14], analysis of AF burden - the percentage of time a patient spends in AF during a monitored period - indicated that LLTS effectively reduces this marker compared to sham stimulation. Notably, the study by Stavrakis et al. [13] reported an 85% lower AF burden at six months in patients who received LLTS compared to those who received sham stimulation. The study by Kulkarni et al. [14] also reported a significant reduction in AF burden compared to patients receiving sham stimulation, demonstrating that LLTS is an effective method for preventing arrhythmia occurrences and reducing the time patients spend in AF episodes.

Furthermore, TNF-α levels were significantly reduced in patients who received LLTS. Specifically, Stavrakis et al. [13] reported a 23% reduction compared to the sham group. Similarly, Stavrakis et al. [12] also observed a significant decrease in TNF-α levels in the LLTS group relative to sham. These findings suggest that LLTS might not only reduce AF burden but also exert beneficial anti-inflammatory effects, which could play a role in mitigating the underlying pathophysiology of AF. Although other cytokines such as IL-10, IL-6, and IL-1β were evaluated in the studies, no significant changes were reported.

Patient responses to LLTS varied, indicating a potential role for predictive biomarkers such as PWA burden [14]; patients with an acute elevation of PWA burden at initial LLTS showed a reduced AF burden at the six-month mark, suggesting that an acute elevation of PWA burden could predict a lower AF burden at follow-up (odds ratio: 0.4; 95% CI: 0.17-0.94; *P* = 0.03), though further research is required to establish PWA burden as a predictive biomarker.

The overall analysis of the studies included in this systematic review demonstrates that, among patients with PAF, LLTS effectively reduces AF burden at the six-month timeframe. These data are important because, although positive outcomes were observed at six months, the chronic nature of AF makes it crucial to assess whether the benefits of LLTS persist beyond this initial timeframe.

It is important to consider the limitations of the studies, including small sample sizes, variability in race and gender, and the relatively short follow-up period of six months. Notably, all studies utilized a stimulation frequency of 20 Hz for one hour, presenting a limitation in assessing treatment response outside this specific dose and timeframe. One of the main limitations noted across studies is the need for continuous monitoring of AF, as none reported the use of continuous ECG recording or wearable devices. As a result, PAF could be absent at the time of measurement if not continuously monitored. It is important to note that not all patients responded to LLTS in the same manner, and no biomarker is currently available to predict which patients will respond best to treatment. Research focused on previously mentioned areas of interest, such as TNF-α and PWA, may help in developing a predictive biomarker and better assessing patient response to LLTS.

Despite the limitations presented in the studies, the results observed suggest that LLTS could become a valuable adjunctive treatment for PAF, particularly in patients who experience frequent episodes or are refractory to conventional therapies, improving parameters such as AF burden and TNF-α levels. Future studies with larger sample sizes, longer follow-up times, and more sophisticated monitoring techniques, such as continuous ECG recording or wearable devices, would provide more robust data on the long-term impact of LLTS and are essential to confirm the findings of this systematic review.

## Conclusions

This systematic review highlights the efficacy of LLTS in suppressing PAF, particularly considering the limited additional treatment options available. While commonly used treatments such as antiarrhythmic drugs and catheter ablation are effective and appropriate for managing the condition, new alternatives like LLTS are emerging as a promising option. The evidence presented in this review shows a significant reduction in AF burden, emphasizing the clinical importance of addressing this arrhythmia given its high recurrence and mortality rates. It is crucial to know that not all patients responded the same way to LLTS, making it evident that further research is required.

Despite the success observed with this treatment, its implementation remains challenging, particularly in resource-limited countries, where access may be restricted by cost, limited awareness of the technique’s availability, and the need for specialized equipment and training. Furthermore, this review identifies several limitations, such as the lack of studies with larger sample sizes and the need for further research into the anti-inflammatory effects of LLTS, specifically TNF-α. However, despite these challenges, integrating LLTS as an adjunctive treatment for AF has the potential to be valuable in cases of PAF refractory to usual treatment and patients with recurrent episodes. Further validation in larger, multicenter randomized trials will be critical to support clinical implementation.
